# Positive Anti–Glomerular Basement Membrane and Proteinase 3–Antineutrophil Cytoplasmic Autoantibody in Infective Endocarditis-Associated Glomerulonephritis: A Diagnostic Challenge

**DOI:** 10.1016/j.ekir.2025.03.030

**Published:** 2025-03-24

**Authors:** Susan J. Thanabalasingam, Ronald A. Booth, Mark Canney, Nataliya Milman, Adrian Agostino, David Massicotte-Azarniouch

**Affiliations:** 1 Division of Nephrology, Department of Medicine, University of Ottawa, Ottawa, Ontario, Canada; 2 Department of Pathology and Laboratory Medicine, The Ottawa Hospital, University of Ottawa, Ottawa, Ontario, Canada; 3 Ottawa Hospital Research Institute, Ottawa, Ontario, Canada; 4 Division of Rheumatology, Department of Medicine, University of Ottawa, Ottawa, Ontario, Canada

## Introduction

Patients with undifferentiated acute kidney injury have broad serologic workups sent when there is suspicion for glomerulonephritis. Nonpathogenic autoantibodies can make interpretation of these tests challenging. Nonpathogenic antineutrophilic cytoplasmic autoantibodies (ANCA) can be seen in many systemic disorders in the absence of ANCA-associated vasculitis (AAV).^1^ Antiglomerular basement membrane (anti-GBM) testing however, is highly sensitive and specific, and nonpathogenic positivity is thought to be rarer.^2^ We report a case of acute kidney injury with positive proteinase 3 (PR3)-ANCA and anti-GBM, both of which were ultimately found to be false positives in the setting of infective endocarditis.

## Case Presentation

A 63-year-old male presented with a 5-day history of vomiting and flank pain and was found to have acute kidney injury. Computed tomography imaging of his abdomen was negative for nephrolithiasis and hydronephrosis. Although his symptoms improved with conservative measures, his kidney injury remained unchanged. His medical history was notable for interstitial lung disease, remote polysubstance use, and treated hepatitis C virus infection. On examination, he had normal vitals, a systolic murmur, and 2+ pitting peripheral edema.

## Results

His creatinine was 296 μmol/l from a baseline of 70 μmol/l 8 months earlier, with an interval serum creatinine of 134 μmol/l 6-weeks before his admission. Blood and urine cultures were negative. Because his urinalysis demonstrated 3+ blood and 2+ protein, a full serologic work-up for glomerulonephritis was sent. His work-up was notable for low C3 (0.80 g/l), low C4 (0.08 g/l), elevated rheumatoid factor (42 kIU/l), and positive antinuclear antibody (1:320), antiPR3 (75 CU [reference range < 20]) ANCA, and anti-GBM (89 CU [reference range < 20]) serologies by immunoassay (INOVA Bioflash, Bedford, MA).

## Discussion

**Figure 1:**
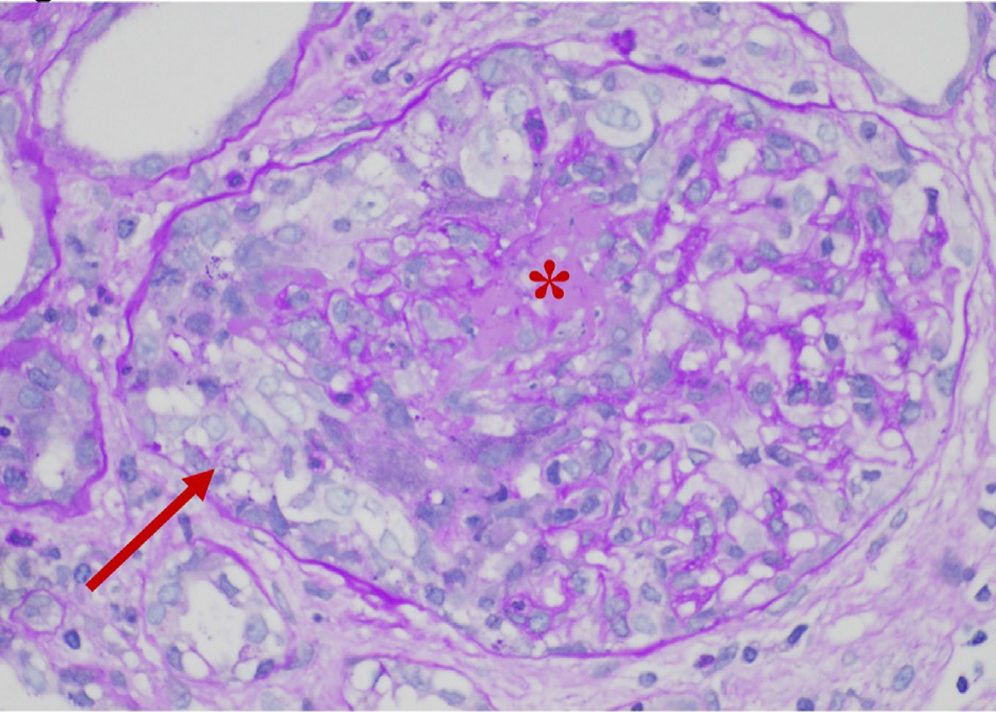
Representative review of a glomerulus with a cellular crescent (arrow) and necrosis (asterisk). 30% to 40% of glomeruli showed fibrocellular crescents with several of them demonstrating fibrinoid necrosis. There was no mesangial or endocapillary proliferation.

Given his positive anti-GBM and antiPR3 serologies, he was empirically treated with i.v. methylprednisolone and the apheresis team was consulted for consideration of plasmapheresis. In the interim, a kidney biopsy and an echocardiogram were performed. His biopsy demonstrated crescentic glomerulonephritis with fibrinoid necrosis of the capillary tuft and a single artery showing necrotizing vasculitis, and moderate fibrosis (Figure 1). Direct immunofluorescence (IF) demonstrated weak staining for IgM, C3, and C1q (Figure S1). The absence of linear IgG staining ruled out anti-GBM disease, and plasmapheresis was not initiated. His echocardiogram however showed aortic and mitral valve vegetations with an aortic root abscess, concerning for endocarditis, although blood cultures were repeatedly negative of antibiotics. Because these biopsy findings can be seen both with AAV and infectious glomerulonephritis due to endocarditis, his case presented a diagnostic dilemma. True AAV triggered by infection also remained a diagnostic possibility. Therefore, methylprednisolone was initially continued, but neither rituximab nor cyclophosphamide were immediately pursued.

With the atypical presentation and diagnostic uncertainty, confirmatory ANCA and anti-GBM testing by indirect IF and alternate immunoassays (Biorad Bioplex, Hercules, CA) were requested using the same serum specimen that was initially positive using the INOVA Bioflash immunoassay. These tests were all negative, suggesting the initial antiPR3 ANCA and anti-GBM tests were indeed false positives. His kidney function stabilized, and once cardiac surgery was scheduled, glucocorticoids were discontinued. He underwent tissue aortic valve repair; intraoperative tissue ultimately returned positive for *Bartonella quintana*. His kidney function improved postoperatively and continued to improve on doxycycline without further immunosuppression, consistent with acute kidney injury caused by infectious glomerulonephritis rather than true AAV. Because his creatinine was 146 μmol/l with bland urine 2 months postoperatively, glomerular disease was felt to be quiescent.

ANCA positivity in the absence of small-vessel vasculitis has been widely reported in several systemic conditions, including autoimmune disease, GI disorders, infection, and drug exposure.^1^ It can be challenging clinically to differentiate nonpathogenic ANCA from AAV triggered by medications or infection where there is ANCA-associated small vessel vasculitis and pauci-immune necrotizing GN. The degree of positivity may be a helpful tool to distinguish patients with AAV from those with nonpathogenic ANCA due to non-AAV diagnoses. A previous study from our center found that antimyeloperoxidase and antiPR3 positive results were significantly higher in patients with AAV than in those with non-AAV conditions.^3^

Anti-GBM immunoassays are highly sensitive and specific, therefore nonpathogenic anti-GBM positivity is thought to be much rarer.^2^ We conducted a systematic literature search using MEDLINE and EMBASE to identify cases of nonpathogenic anti-GBM, focusing on concurrent ANCA positivity. Of 42 abstracts screened, 17 studies were assessed for eligibility, and 13 were included (Table S1, Supplementary References). Anti-GBM false positivity with concurrent nonpathogenic ANCA (*n* = 5) was reported in the setting of infection, lupus, and nonspecific reactions to nonantigenic substances. A single study reported nonpathogenic anti-GBM in the context of biopsy-proven myeloperoxidase-AAV.^4^ Kidney biopsies were performed in most cases (*n* = 8 [62%]) to confirm nonpathogenic anti-GBM positivity.

Among the included studies, patients in a minority of cases received immunosuppression, including steroids (*n* = 5 [38%]) and plasmapheresis (*n* = 2 [15%]). Similarly, 39% of cases in a previous review of ANCA positivity in endocarditis received concurrent immunosuppression with antibiotics.^5^ Immunosuppression was primarily administered when there was persistent kidney function deterioration with active urine despite antimicrobials, where AAV triggered by infection was suspected.^5^ Repeat kidney biopsy to assess for ongoing glomerular inflammation despite treating endocarditis appropriately with antibiotics or surgery may be helpful to distinguish infectious glomerulonephritis from true AAV triggered by infection.

Testing for ANCA and GBM antibodies can be performed by both indirect IF and immunoassay. Indirect IF involves antibody detection using human granulocytes and primate kidneys, respectively. This methodology is semiquantitative and requires expertise in interpretation. Immunoassays, however, use a purified antigen to quantitatively measure antibodies, requiring less expertise, but can suffer from false positive cross-reactivity. The 2017 international consensus on ANCA testing has established immunoassays as the most appropriate first-line test.^6^

Given that the initial false positives in our case involved immunoassays, we suspect that the immune response to systemic infection with endocarditis led to cross-reactivity, which produced the false positive antibody results. This is in line with a hypothesis from a previous case report that similarly found PR3-ANCA and anti-GBM false positivity in the setting of *Staphylococcus capitis* infective endocarditis.^7^ Notably, the antigens used in immunoassays are not standardized, and assays from different manufacturers use different antigens for antibody detection.^8^ This explains why repeat testing on the same serum sample using alternate immunoassays from other centers were negative in our case.

Although confirmatory IF was negative in our case, it is important to note that pathogen-specific associations with positive ANCA using indirect IF have been reported with *Bartonella* specifically. In a series of 25 patients with *Bartonella* endocarditis, the 60% prevalence of positive ANCA by IF was postulated to be related to high degrees of chronic vascular inflammation with *Bartonella* resulting in autoantibody production.^9^

Because there can be substantial overlap between the clinical manifestations of systemic processes such as endocarditis and vasculitis, diagnosis can be difficult, and nonpathogenic autoantibodies and false positives pose further diagnostic challenges (Table S2). Distinguishing between infection and vasculitis is crucial to avoid exposing patients to unnecessary immunosuppression (Table 1). Discussing this case with our clinical biochemists and pathologists added tremendous value; the fairly low-level initial antibody positivity, along with atypical presentation, led to suspicion of false positivity and triggered alternate testing methods. These confirmed false positivity, which altered disease management and spared the patient long-term immunosuppression. We therefore strongly recommend communicating directly with biochemists and pathologists when the clinical picture does not align with the test results to help guide interpretation, further testing and management.

**Table 1:** Teaching points

• It is vital to consider infection in the differential diagnosis of AAV and send infectious work-up when the clinical picture is suspicious or has atypical features to avoid exposing patients to potentially harmful immunosuppression.
• Nonpathogenic autoantibodies and false positives can be clinically challenging to discern because manifestations of endocarditis and vasculitis can overlap significantly.
• When the clinical picture does not align with the results of the serologic work-up or biopsy, directly liaising with clinical biochemists and pathologists can be paramount.

AAV, antineutrophil cytoplasmic autoantibody (ANCA)-associated vasculitis.

## Disclosure

All the authors declared no competing interests.

## Patient Consent

The authors declare that they have obtained consent from the patient discussed in the report.

## Supplementary Material

Supplementary File (PDF) **Supplementary References. Figure S1.** Immunofluorescence from kidney biopsy. **Table S1.** Nonpathogenic anti-GBM testing. **Table S2.** Primary AAV versus GN in IE with positive ANCA.

## Footnotes

Supplementary File (PDF)

**Supplementary References.**
**Figure S1.** Immunofluorescence from kidney biopsy. **Table S1.** Nonpathogenic anti-GBM testing. **Table S2.** Primary AAV versus GN in IE with positive ANCA.
