# Supplementary material for: Positive Anti–Glomerular Basement Membrane and Proteinase 3–Antineutrophil Cytoplasmic Autoantibody in Infective Endocarditis-Associated Glomerulonephritis: A Diagnostic Challenge
Source: Kidney Int Rep. 2025 Mar 24;10(6):2062–4. doi: 10.1016/j.ekir.2025.03.030 (PMC12230994; doi:10.1016/j.ekir.2025.03.030)
Supplement: Supplementary File (PDF) — Supplementary References. Figure S1. Immunofluorescence from kidney biopsy. Table S1. Nonpathogenic anti-GBM testing. Table S2. Primary AAV versus GN in IE with positive ANCA. [file mmc1.pdf]

Table S1. Non-pathogenic anti-GBM testing

**Study ID, Author, Year**	**Anti-GBM Test Level (Ref Range)**	**Concurrent ANCA**	**Clinical condition**	**Gold standard confirmatory test**	**Outcome**	**Immunosuppression**
#1, Yoshida, 2024	FEIA* EliA GBM Well 70.4 IU/mL (0-6.9)	Negative	• Vancomycin-induced ATN<br>• Reaction to bovine serum albumin	• Kidney biopsy: acute tubular necrosis (ATN), negative IF<br>• CEIA negative<br>• FEIA with GBM antigen removed positive	Cr 3.2 mg/dL POD10 → 0.84 POD43	Prednisolone 30 mg/d x 4d
#2, Sato, 2023	MEBLux CEIA** 13.1 U/mL (<3)<br><br>Repeats 11.4-30.5 U/mL	Negative	Non-specific reaction	• FEIA < 1.5 U/mL<br>• ELISA 4.39 U/mL (<20)<br>• Immunoblotting and immunohistochemistry negative	Intermittent hematuria	None
#3, Zijlstra, 2018	FEIA 9.7 (Day 1) → 16 (Day 4) kIU/l (<7 negative, equivocal 7–10)	Negative	Acute Hantavirus Infection	N/A	Cr 305 → 72 umol/L 12 w post-discharge EIA < 0.8	None
#4, deJoode, 2014	Dotblot n=2<br><br>ELISA*** n=1	Anti-MPO	MPA	Kidney biopsy not consistent with anti-GBM	Data not provided	None
#5, Hernandez, 2006	40 U/mL (<5)	Anti-MPO 9 U/mL (<7 negative, 7-15 equivocal)	TB reactivation in a patient with HIV	Kidney biopsy: immune complex glomerulonephritis with granular IF	KRT initiation 3-years post presentation	None

#6, Ruster, 2006	Imtec ELISA >200 U/mL	Negative	• Volume depletion and acute interstitial nephritis due to NSAIDs<br>• Reaction to bovine serum albumin	• Kidney biopsy: ATN, interstitial nephritis, negative IF<br>• Negative IF using monkey renal slides<br>• Orgentec ELISA and Western Blot negative	Normal renal function 4 months later	Methylprednisolone and plasmapharesis initially
#7, DeAangelo, 2002	Quest ELISA 15 EU/mL	Negative	Diabetic nephropathy with interstitial nephritis	Kidney biopsy: no crescents, interstitial nephritis, with linear IgG and albumin deposition	Cr stabilized at 5.0 mg/dL without further KRT	Methylprednisolone → oral prednisone
#8, Jaskowski, 2002	INOVA n = 2 (28.4 units [0-20])<br><br>Binding Site n = 7 (7.3 U/mL ([0-3])<br><br>Scimedx n = 8 (11.2 EU/mL [0-5])<br><br>Wieslab n = 2 (157 units [<10 negative, 10-20 equivocal])	Negative	N/A	GBM immunofluorescence assay (IFA) using tissue from human kidney	N/A	None

#11, Savige, 1994	ELISA n = 18	ANCA ELISA n = 12	HIV	Specificity of binding was tested using inhibition studies in n =4, all were negative	N/A	None
#32, Buhl, 2017	200 U/mL	Anti-PR3 Anti-MPO	Reaction to blocking agent	Negative when repeated using another producer's tests	N/A	None
#12, Yamada, 2018	11.9 U/mL	PR3 ANCA 4.1 U/mL	SLE with pulmonary hemorrhage	Kidney biopsy: ATN, granular IF	Cr 1.03 mg/dL → normal	Methylprednisolone → oral prednisolone and plasmapharesis
#34, Manley, 2016	56 CU	Negative	Hepatitis C	Kidney biopsy: membranoproliferative glomerulonephritis, granular IF	Improved Cr with hematuria resolution	Methylprednisolone → course of high dose steroids
Chiba, 2022	29 U/mL (<7)	PR3 ANCA 63.9 IU/mL (<3.5)	Staphylococcus capitis IE	Kidney biopsy: focal crescentic sclerosing glomerulonephritis with C3c mesangial deposition on IF	Improved Cr	None

**FEIA = Fluorescent enzyme immunoassay, **CEIA = Chemiluminescent enzyme immunoassay, ***ELISA = enzyme-linked immunosorbent assay*

Table S2. Primary-AAV vs. GN in IE with positive ANCA

**Primary AAV**	**GN in IE with positive ANCA**
**Clinical presentation**: There is substantial overlap with respect to clinical manifestations and findings on exam between primary AAV and GN in IE. Cardiac involvement is notably rare in primary AAV.	**Clinical presentation**: Valvulopathy and its consequences are far more common in the setting of GN in IE.
**Lab testing**: Anti-MPO positivity is more commonly seen in GN from primary AAV. The degree of antibody positivity tends to be higher in patients with primary AAV than in those with GN in IE with positive ANCA.	**Lab testing:** Anti-PR3 positivity is most commonly seen in GN in IE, and dual positivity for anti-MPO and anti-PR3 can also been seen.

**Kidney biopsy:** Crescentic lesions with necrosis on light microscopy, and pauci-immune immunofluorescence.	**Kidney biopsy:** Crescentic lesions with endocapillary hypercellularity on light microscopy. IgM dominant and C3 staining can be seen on immunofluorescence, but it may also be pauci-immune. Endocapillary proliferation and deposits on immunofluorescence and electron microscopy are common.

Figure S1.

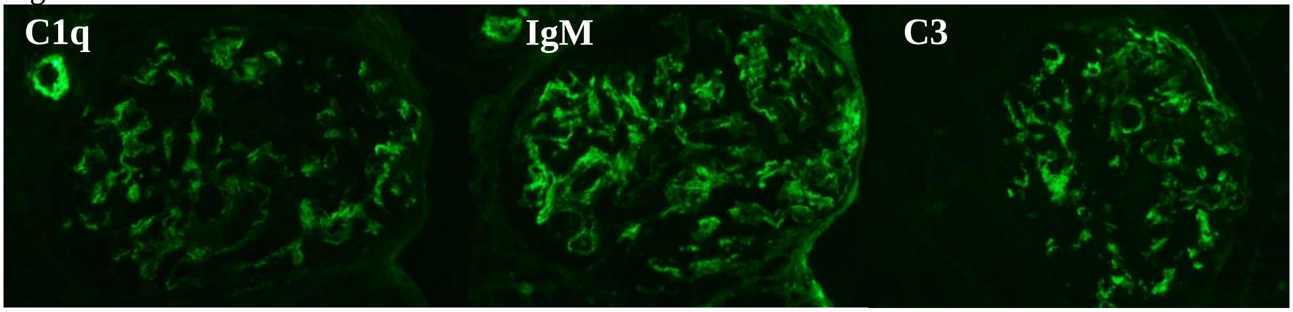

There was trace segmental mesangial and capillary staining for C1q, IgM C3, lambda and kappa. Electron micropscopy revealed only very subtle electron dense deposits in a few mesangial areas, which was felt to be consistent with a pauci-immune GN.

Supplementary References:

S1. Yoshida R, Azegami T, Yamaguchi S, et al. False-positive Serum Antiglomerular Basement Membrane Antibody due to Bovine Serum Albumin-containing Surgical Adhesive: A Case Report. *Kidney Med*. 2024;6(10):100880. doi:10.1016/j.xkme.2024.100880

S2. Sato M, Nishibata Y, Masuda S, et al. Demonstration of equivocal anti-glomerular basement membrane antibody positivity as a non-specific reaction through multiple immunologic assays in a case of pediatric asymptomatic hematuria. *Clin Biochem*. 2023;120(September):110650. doi:10.1016/j.clinbiochem.2023.110650

S3. Zijlstra HW, Mulder AHL, Geeraedts F, Visser F. Falsely positive anti-glomerular basement membrane antibodies in a patient with hantavirus induced acute kidney injury - A case report. *BMC Nephrol*. 2018;19(1):1-4. doi:10.1186/s12882-018-1082-3

S4. De Joode AAE, Roozendaal C, Van Der Leij MJ, Bungener LB, Sanders JSF, Stegeman CA. Performance of two strategies for urgent ANCA and anti-GBM analysis in vasculitis. *Eur J Intern Med*. 2014;25(2):182-186. doi:10.1016/j.ejim.2013.11.011

S5. Hernandez GT, Critchfield JM, Rodriguez RA. Interpretation of serologic tests in an HIV-infected patient with kidney disease. *Nat Clin Pract Nephrol*. 2006;2(12):708-712. doi:10.1038/ncpneph0324

S6. Rüster M, Kiehntopf M, Gröne HJ, Wolf G. A Friday afternoon case of apparent anti-glomerular basement nephritis. *Nephrol Dial Transplant*. 2006;21(8):2328-2330. doi:10.1093/ndt/gfl220

S7. DeAngelo AJ, Lancaster-Weiss KJ, Eliason D, Troyer D, Wortham WG. Diabetic nephropathy with interstitial nephritis presenting with a false-positive anti-GBM antibody. *Clin Nephrol*. 2002;57(5):381385.

S8. Jaskowski TD, Martins TB, Litwin CM, Hill HR. Comparison of four enzyme immunoassays for the detection of immunoglobulin G antibody against glomerular basement membrane. *J Clin Lab Anal*. 2002;16(3):143-145. doi:https://dx.doi.org/10.1002/jcla.10034

S9. Savige JA, Chang L, Horn S, Crowe SM. Anti-nuclear, anti-neutrophil cytoplasmic and anti-glomerular basement membrane antibodies in hiv-infected individuals. *Autoimmunity*. 1994;18(3):205-211. doi:10.3109/08916939409007997

S10. Buhl D. A rare case report of a polyspecific serum. *Clin Chem Lab Med*. 2017;55(Supplement 1):S332-. doi:https://dx.doi.org/10.1515/cclm-2017-5007

S11. Yamada T, Mugishima K, Higo S, et al. A case of anti-glomerular basement membrane antibody-positive systemic lupus erythematosus with pulmonary hemorrhage successfully treated at an early stage of the disease. *J Nippon Med Sch*. 2018;85(2):138-144. doi:10.1272/jnms.2018_85-21

S12. Manley P, Ismail I, Francis L, Venuthurupalli S. Membranoproliferative Glomerular Nephritis (MPGN) in a patient with hepatitis C, cryoglbulinaemia and falsely-positive anti-GBM antibodies. *Nephrology*. 2016;21(Supplement 2):272-273. doi:https://dx.doi.org/10.1111/nep.12888

S13. Chiba Y, Takahashi K, Makino R, et al. Glomerulonephritis Associated with Infective Endocarditis Showing Serological Positivity for PR3-anti-neutrophil Cytoplasmic Antibody and Anti-glomerular Basement Membrane Antibody. *Intern Med*. 2022;61(14):2179-2185. doi:10.2169/internalmedicine.8385-21
